# Genetic Variability in Vitamin D Receptor and Migraine Susceptibility: A Southeastern European Case-Control Study

**DOI:** 10.3390/neurolint15030069

**Published:** 2023-09-05

**Authors:** Maria Papasavva, Michail Vikelis, Vasileios Siokas, Martha-Spyridoula Katsarou, Emmanouil V. Dermitzakis, Athanasios Raptis, Efthimios Dardiotis, Nikolaos Drakoulis

**Affiliations:** 1 Research Group of Clinical Pharmacology and Pharmacogenomics, Faculty of Pharmacy, School of Health Sciences, National and Kapodistrian University of Athens, 157 71 Athens, Greece; 2 Headache Clinic, Mediterraneo Hospital, 166 75 Glifada, Greece; 3 Laboratory of Neurogenetics, Department of Neurology, University Hospital of Larissa, Faculty of Medicine, School of Health Sciences, University of Thessaly, 411 00 Larissa, Greece; 4 Euromedica General Clinic, 546 45 Thessaloniki, Greece

**Keywords:** VDR, precision medicine, single nucleotide variants (SNVs), primary headaches, migraine genetics, *FokI*, *TaqI*, *BsmI*

## Abstract

Migraine is a common primary headache disorder with both environmental and genetic inputs. Cumulative evidence indicates an association between vitamin D and headache. Unravelling the precise role of vitamin D and its receptor in the pathophysiology of migraine can eventually contribute to more efficient prevention and management of this headache disorder. The aim of the study was to investigate the relation of the three most studied *VDR* variants, i.e., *FokI* (rs2228570), *TaqI* (rs731236) and *BsmI* (rs1544410), with migraine susceptibility and distinct clinical phenotypes in a Southeastern European case-control population residing in Greece. DNA was extracted from 191 unrelated patients diagnosed with migraine and 265 headache-free controls and genotyped using real-time PCR (LightSNiP assays) followed by melting curve analysis. Genotype frequency distribution analysis of the *TaqI* and *BsmI* variants showed a statistically significant difference between migraine cases and controls. In addition, subgroup analyses revealed a significant association between all three studied *VDR* variants, particularly with a migraine without aura subtype. Therefore, the current study provides supporting evidence for a possible association of *VDR* variants with migraines, particularly migraine without aura susceptibility in Southeastern Europeans residing in Greece, further reinforcing the emerging role of vitamin D and its receptor in migraines.

## 1. Introduction

Migraine is a common primary headache disorder with a high disability burden and considerable detrimental effects on public health [1]. According to the Global Burden of Disease Study 2019 (GBD2019), migraine ranks second among the causes of global years lived with disability (YLDs), being responsible for 4.8% (0.8–10.1) of total YLDs and comprising 88.2% (60.7–97.7) of the burden of headache disorders in 2019. The highest burden, i.e., 7.3% (1.1–15.1), was observed in the age group 15–49 years, the most productive years of life in both genders [2]. Although the pathophysiology of the disease has not been fully elucidated, among the proposed mechanisms being implicated are the activation of the trigeminovascular system, cortical spreading depression, inflammation and vascular dysfunction [3]. Besides environmental factors, migraine is largely affected by genetic factors, with several genetic variants, each having a minor effect contributing to its liability [4,5,6].

In the last decades, vitamin D, a fat-soluble hormone belonging to the secosteroid family, has received enormous attention due to its large spectrum of musculoskeletal and non-skeletal biological functions, including regulation of cellular proliferation and differentiation, hormone secretion, control of immune function and metabolism [7]. A two-step sequentially metabolism of vitamin D occurs to attain its biological effects; the first step is the metabolism in the liver by the enzyme D-25-hydroxylase (CYP2R1) into 25-hydroxyvitamin D (25(OH)D), the major circulating form, followed by hydroxylation into 1α,25-dihydroxyvitamin D (1,25(OH)_2_D) by 1-alpha-hydroxylase (CYP27B1) located in target organs, e.g., the kidney, skin, brain, lungs, eyes, and breasts. 1,25(OH)_2_D, the biologically active metabolite of vitamin D, exerts its responses through the vitamin D receptor (VDR) via genomic and non-genomic functions [8,9,10]. VDR occurs ubiquitously in almost all cells and tissues, and approximately 3% of the human genome is regulated by VDR-1,25(OH)_2_D [11]. The activity of VDR and its ligand do not fully overlap. VDR can also bind its low-affinity nutritional ligands, such as curcumin and polyunsaturated fatty acids, while alternative molecules, e.g., resveratrol, can promote the nuclear VDR signalling [12,13]. Single nucleotide variants (SNVs) in the VDR gene, which is located in chromosome 12 (12q13.11), can likely modify VDR expression and function [14,15]. Among the most studied *VDR* SNPs are *FokI* (rs2228570), *TaqI* (rs731236) and *BsmI* (rs1544410). The *Fokl* (rs2228570) polymorphic variant is a T (or *f* allele) to C (or *F* allele) substitution at the *VDR* translation start site. The presence of the C allele eliminates the translation start site in exon 2, resulting in a shortened protein by three amino acids, which exerts higher transcriptional activity [14,16,17,18]. In a longitudinal population-based study, the T allele was associated with a higher 25(OH)D level [16]. The *BsmI* (rs1544410) variant, a G (or b allele) to A (or B allele) substitution in intron 8 and the *TaqI* (rs731236) variant, a T (or T allele) to C (or t allele) substitution in exon 9, both located at the 3′ end of the *VDR*, may modify transcript stability [14,19,20].

A review by Prakash et al. provided evidence for a positive association between prevalence rates of both migraine and tension-type headache (TTH) and higher latitudes. In addition, the review pointed out that headache attacks are more frequent during autumn and winter and less prevalent during summer. Since the aforementioned observations are in accordance with regional and seasonal alterations in serum vitamin D levels, the increasing prevalence of these headache disorders seems to be related to vitamin D deficiency [21]. Likewise, a study by Mitsikostas et al. in Greece indicated that daily headache prevalence might be affected by both latitude and low mean temperature [22]. Several published scientific articles addressed the relationship between vitamin D and headache, with most of them indicating an inverse association between migraine and serum vitamin D levels [23,24,25,26,27,28,29,30,31,32,33,34,35,36,37,38,39,40]. In addition, serum VDR levels were found to be lower in migraine patients compared to controls [35]. Further to the wide expression of vitamin D receptor (VDR) and vitamin D key metabolic enzymes, including 25-hydroxylase, 1-alpha-hydroxylase (CYP27B1) and CYP24A1 in Central Nervous System (CNS) regions, vitamin D seems to be involved in various physiological brain processes, i.e., brain development, synaptic plasticity, neurotransmission, and cell death prevention [25,41,42,43]. SNVs in the vitamin D pathway genes, including *VDR*, *CYP2R1*, *CYP24A1* and *CYP27B1*, were associated with vitamin D serum levels [44,45,46]. Although the exact role of vitamin D in migraine remains obscure, vitamin D might be implicated via a variety of proposed mechanisms in the complex pathways involved in the pathophysiology of migraine [47,48,49,50].

While several prophylactic medications and pharmacological treatments alleviating migraine acute attacks are available [51], the quality of life of a great percentage of migraine patients is still declining due to improper diagnosis and/or therapeutic treatment. Thus, new diagnostic and therapeutic treatment strategies are needed to establish precision-medicine approaches, prevent migraine progression, attenuate disabilities related to the disorder and improve patient’s quality of life. Since VDR has a central role in exerting the majority of 1,25(OH)_2_D biological responses, and it occurs in various CNS regions, VDR could represent a candidate gene for migraine. The implication of VDR genetic variants in migraine pathogenesis has not been previously investigated in the Southeastern European population residing in Greece, although a study in an Iranian population provided evidence for an association between *VDR* polymorphisms and migraine susceptibility [52]. Hence, the aim of the current study was to investigate the possible association of three variants in the gene encoding for vitamin D receptor (*VDR*), namely rs2228570 (*FokI*), rs731236 (*TaqI*) and rs1544410 (*BsmI*), with migraine susceptibility and clinical phenotypes, in a Southeastern European case-control population residing in Greece. The findings of the current study may eventually shed more light on the relationship between vitamin D and migraine and contribute to the identification of molecules involved in disease pathophysiology, the discovery of new therapeutic targets, the establishment of migraine-specific biomarkers for precision medicine strategies, and to overcome the barriers in the treatment of migraine.

## 2. Subjects and Methods

### 2.1. Study Population

A total of 191 migraine subjects (33 males and 158 females) aged between 18 to 72 years (mean ± standard deviation 42.0 ± 11.5 years) were prospectively recruited in specialised headache clinics located in Glyfada and Thessaloniki, Greece, from September 2019 to July 2021 as a case group. The migraine diagnosis was made by experienced headache specialists according to the International Classification of Headache Disorders 3rd edition (ICHD-3) guidelines. Key inclusion criteria included age ≥ 18 years; clinically diagnosed migraine [1.1 migraine without aura (MwoA); 1.2 migraine with aura (MwA); or 1.3 chronic migraine (CM)]; and Southeastern European origin. A group of 265 headache-free subjects (133 males and 132 females), aged between 21 to 85 years (mean ± standard deviation 57.7 ± 12.8 years), was recruited from the Neurology Department, University Hospital of Larissa, Greece and served as a control group.

A written informed consent was provided by all study subjects. The study was approved by the appropriate Ethics Committees (Mediterraneo Hospital, Glyfada, Greece, and University Hospital of Larissa) and conducted according to the principles outlined in the Declaration of Helsinki.

### 2.2. DNA Extraction and Genotyping

Epithelial cell samples were collected from the oral cavity of each subject. Sterile buccal swabs were used for the collection. For the DNA extraction, a commercial nucleic acid isolation kit (Nucleospin Tissue; Macherey-Nagel GmbH & Co., KG, Düren, Germany) was used, according to the manufacturer’s protocol. All extracted DNA samples were stored at −20 °C until further analysis. Genotyping of the three investigated *VDR* variants i.e., *FokI* (rs2228570), *TaqI* (rs731236), and *BsmI* (rs1544410), was carried out by real-time Polymerase Chain Reaction (LightCycler^®^ 480; Roche) using simple probes for each SNP (LightSNiP Assays; TIBMOLBIOL, Berlin, Germany) according to the manufacturer’s instructions. DNA samples (50 ng) were amplified using the respective LightSNiP Assay and LightCycler FastStart DNA Master HybProbe Mix (Roche, Germany). The following PCR protocol was applied: initial denaturation at 95 °C for 10 min, followed by 45 cycles of denaturation at 95 °C for 10 s, annealing at 60 °C for 10 s and elongation at 72 °C for 15 s. Melting curve analysis was performed to determine homozygosity for the wild-type alleles, heterozygosity, and homozygosity for the variant alleles.

### 2.3. Statistical Analysis

Categorical data are presented as frequencies (n) and percentages (%), and continuous data as mean ± standard deviation (SD). Differences in genotypic and allelic frequency distribution between case and control subjects and between case subgroups were evaluated using chi-square (χ^2^) (Pearson or Fischer’s exact) tests. Crude odds ratios (OR) with their corresponding 95% confidence intervals (95% CI) were calculated to investigate the association of the selected *VDR* variants with migraine susceptibility and clinical aspects under co-dominant, dominant, recessive, over-dominant genotypic and allelic inheritance models. To exclude any bias introduced by the differences in age and sex ratio between the study groups, adjustment for potential confounding factors, including age and sex, as well as Body Mass Index (ΒΜΙ) and smoking status, was performed using logistic regression analysis. Kolmogorov–Smirnov and Shapiro–Wilks tests were used to examine the distribution of the continuous variables. Non-parametric test. i.e., Kruskal–Wallis was used to investigate the association of the three *VDR* variants with disease-specific clinical characteristics in the case subjects (disease age at onset and frequency of migraine attacks), while chi-square test and logistic regression analysis were used to assess the association of the variants with typical duration of migraine attacks (≤24 h vs. >24 h). All statistical tests were two-sided, and a *p*-value less than 0.05 was considered statistically significant. Statistical analyses were carried out using IBM SPSS Statistics software (version 28.0 for Windows). The consistency with Hardy–Weinberg Equilibrium (HWE) for *FokI* (rs2228570), *TaqI* (rs731236) and *BsmI* (rs1544410) variants in the control group was verified (*p* > 0.05) using the web-based Online Encyclopedia for Genetic Epidemiology studies software [53]. Haplotype analysis was performed using the SHEsis web-based platform (http://analysis.bio-x.cn/myAnalysis.php, accessed on 4 June 2023) [54,55]. An a posteriori power analysis was performed using G-Power software [56], which resulted in a 0.999 power for the chi-square test (degrees of freedom = 2). Thus, it confirms that the study results/conclusions were reliable and robust.

## 3. Results

### 3.1. Demographic and Clinical Characteristics

The population of the current prospective, case-control study consisted of 456 non-related subjects (191 migraine patients and 265 headache-free controls) of Southeast European origin residing in the geographical area of Greece. Detailed information on demographics and clinical disease-specific characteristics was obtained for each case subject via predesigned questionnaires. One hundred and nine (109) case subjects met the diagnostic criteria for MwoA (57.1%), 24 for MwA (12.6%) and 58 for CM (30.4%). The mean ± SD age of disease onset was 20.0 ± 8.4 years, ranging from 5 to 52 years. Positive family history was reported for 137 migraine patients (71.7%). Data collected from control subjects included only age and sex (Table 1).

### 3.2. Associations between VDR SNVs and Migraine Phenotypes

As presented in Table 2, the genotype frequency distribution for the *TaqI* (rs731236) and *BsmI* (rs1544410) variants differed significantly between migraine and control subjects. Heterozygosity for both *TaqI* [TC vs. TT + CC: OR_adj_ (95%CI) = 1.697 (1.053–2.733), *p*_adj_ = 0.030] and *BsmI* [GA vs. GG + AA: OR_adj_ (95%CI) = 1.611 (1.002–2.592), *p*_adj_ = 0.049] variants was significantly more prevalent in migraine subjects compared to controls and remained significant after adjustment for age and sex. Regarding the *FokI* variant, no statistically significant differences were observed between case and control subjects in any of the genetic inheritance models assessed (*p* > 0.05) (Table 2).

Genotype frequency distribution analysis between migraine subtypes and control subjects revealed significant associations for all three investigated *VDR* variants with MwoA susceptibility. Homozygosity for the less common alleles of the *TaqI* [CC vs. TC + TT: OR_adj_ (95%CI) = 0.427 (0.183–0.997), *p*_adj_ = 0.049; CC vs. TC: OR_adj_ (95%CI) = 0.378 (0.157–0.908), *p*_adj_ = 0.030] and *BsmI* [AA vs. GA + GG: OR_adj_ (95%CI) = 0.399 (0.170–0.934), *p*_adj_ = 0.034; AA vs. GA: OR_adj_ (95%CI) = 0.374 (0.156–0.898), *p*_adj_ = 0.028] variants was significantly less prevalent in MwoA case subjects compared to control subjects after adjusting for age and sex. Concerning the *FokI* variant, χ^2^ test showed a significant difference between MwoA and controls [CT vs. CC + TT: OR (95%CI) = 1.663 (1.061–2.605), *p* = 0.026; CC vs. CT: OR (95%CI) = 0.621 (0.387–0.999), *p* = 0.049], and the significance remained after adjusting for sex, while disappeared when adjusting for both age and sex (Table 3). No significant differences in the genotype and allele frequency distributions were observed between MwA patients versus controls, CM patients versus controls or MwoA versus MwA patients (*p* > 0.05).

The frequency distribution analysis of the *VDR* haplotypes in migraine patients versus the control group (Table 4) and MwoA patients versus controls (Table 5) did not reveal any significant associations.

Furthermore, frequency distribution analysis of the three investigated variants in subsets of migraineurs according to migraine attack duration (≤24 h vs. >24 h) indicated no significant differences. Finally, no significant association was shown for any of the *VDR* variants studied with disease-specific clinical features, i.e., age at onset and attack frequency in the study cohort (*p* > 0.05) (Table 6).

## 4. Discussion

To the best of the author’s knowledge, the current case-control study is the first investigating the association of the three most intensively studied SNVs in *VDR*, namely *FokI* (rs222857, also known as rs10735810), *TaqI* (rs731236), and *BsmI* (rs1544410), with the susceptibility to develop migraine and diverse clinical phenotypes and features in a Southeastern European population residing in Greece. According to the genotypic and allelic frequency distribution analysis, although no significant association for the *FokI* variant with the occurrence and development of migraine was revealed in the study cohort, heterozygous TC (*p_adj_* = 0.030) and GA (*p_adj_* = 0.049) genotypes for the *TaqI* and *BsmI* variants, respectively, were significantly more prevalent in migraine cases compared to control subjects. Additionally, subgroup analysis revealed an association between all studied *VDR* variants and the MwoA subtype. Consequently, variability in the *VDR* gene may serve as a genetic susceptibility factor for migraine and MwoA subtype in Southeastern Europeans.

Scientific data point toward a key role of vitamin D in brain health maintenance. VDR and vitamin D metabolising enzymes are present in various brain regions, indicating the distinctive functioning of vitamin D and particularly VDR in the CNS. In addition, evidence indicates that vitamin D plays a crucial role in brain development, acts as a neuroprotective factor by controlling neurotrophic factor production, influences the release of several neurotransmitters, such as serotonin and dopamine, and serves as a potent antioxidant agent [50]. Migraine is a complex brain disorder with metabolic, hormonal, and genetic components. While multiple lines of evidence highlight a link between migraine headaches and vitamin D, with various studies denoting vitamin D deficiency or insufficiency in a great percentage of migraine sufferers, the precise relation between migraine and vitamin D deficiency remains enigmatic [38,57].

Variability in the *VDR* gene may modify VDR expression, structure, or function and, therefore, influence vitamin D signalling pathways [58]. To date, only two studies have investigated the association between *VDR* SNVs and migraine susceptibility in diverse populations. A previous study by Motaghi et al. indicated an association of *TaqI* and *FokI* SNVs with MwoA in an Iranian case-control population. Heterozygous genotypes for both *FokI* (33.9% vs. 15%, p = 0.001) and *TaqI* (50.4% vs. 36%, *p* = 0.018) variants were statistically more prevalent in MwoA patients compared to control subjects [52]. In accordance, the heterozygosity for *FokI* (54.1% vs. 41.5%, *p* = 0.026) and *TaqI* (54.1% vs. 42.6%, *p* = 0.043) SNVs were also more frequent in MwoA patients compared to headache-free controls in the population of the current study. On the contrary, a study by Schürks et al. investigating the relationship between 77 SNVs and migraine in a Caucasian female population with self-reported migraine found no significant association of the *VDR FokI* and *BsmI* variants with migraine [59]. A major difference between the current study and the study by Schürks et al. is the population selection; Schürks et al. included only female U.S. Caucasian health professionals aged ≥ 45 years participating in the Women’s Health Study with self-reported migraine and migraine aura status, whereas the current study included both male and female migraine patients residing in Greece, diagnosed by experienced headache specialists according to the International Classification of Headache Disorders 3rd edition (ICHD-3) guidelines.

The study has certain potential limitations that should be considered when interpreting the current findings. Firstly, a limitation of the study is the relatively small sample size, which has insufficient power to detect associations with small effect size genetic variants; migraine is mainly a polygenic disorder with several genetic variants, each having a small effect size. Hence, genetic variants with small effect sizes do occur. Although adjustment for sex, age and other confounding factors was performed, the difference in sex ratio between cases and controls and the small number of male subjects in the case group due to the female preponderance of the disorder may serve as a potential limitation of the study. Moreover, investigation of other variants in the *VDR* gene and in further genes encoding for proteins implicated in vitamin D signalling systems, such as CYP2R1, CYP27B1, and CYP24A1, was not conducted, restricting the acquisition of additional genetic information. Finally, other confounding factors, including gene–gene or gene–environment interactions, were not assessed. Therefore, larger-scale studies from diverse ethnic populations are required to obtain more definite results.

## 5. Conclusions

In conclusion, the findings of the current study further support a possible association of SNVs in *VDR* with the susceptibility to develop migraine and MwoA subtype. Heterozygosity for the *VDR TaqI* (TC) and *BsmI* (GA) variants may serve as a risk factor for migraine and MwoA susceptibility in the studied Southeastern European population. Despite the abovementioned limitations, the current study provides a reference for further investigations among the Southeastern European population to translate genetically derived data into clinical applications for the precision management of migraines.

## Figures and Tables

**Table 1:** Demographic and clinical characteristics of the study population.

	Migraine Patients (N = 191)		Headache-Free Controls (N = 265)	
**Age** (years) *	42.0 ± 11.5 ranged from 18 to 72		57.7 ± 12.8 ranged from 21 to 85	
**Sex**, *n (%)*				
Male	33	(17.3)	133	(50.2)
Female	158	(82.7)	132	(49.8)
**BMI** (kg/m^2^) *	24.6 ± 4.2		-	
**Smoking**, *n (%)*				
Never	116	(60.7)	-	
Former	23	(12.1)	-	
Ever	52	(27.2)	-	
**Age of onset** (years) *	20.0 ± 8.4 ranged from 5 to 52		-	
**Positive family history**, *n (%)*	137	(71.7)	-	
**Type of Migraine**, *n (%)*				
1.1 MwoA	109	(57.1)	-	
1.2 MwA	24	(12.6)	-	
1.3 CM	58	(30.4)	-	

* Values are presented as mean ± SD, BMI, body mass index; MwoA, Migraine without Aura; MwA, Migraine with Aura; CM, Chronic Migraine.

**Table 2:** Genotypic and allelic frequency distribution analysis of the *VDR* SNPs between migraine cases and controls.

	Migraine Cases (N = 191)		Controls (N = 265)		OR (95%CI)	*p*	OR_adj_ (95%CI) *	*p_adj_* *
	*n*	*(%)*	*n*	*(%)*				
**FokI** **rs2228570**								
CC	80	(41.9)	123	(46.4)	1.0 (reference)	-	-	-
CT	95	(49.7)	110	(41.5)	0.753 (0.508–1.116)	0.157	0.891 (0.540–1.470)	0.651
TT	16	(8.4)	32	(12.1)	1.301 (0.670–2.524)	0.436	0.988 (0.400–2.443)	0.980
CT + TT	111	(58.1)	142	(53.6)	0.832 (0.572–1.211)	0.337	0.899 (0.559–1.445)	0.659
TT	16	(8.4)	32	(12.1)	1.0 (reference)	-	-	-
CT	95	(49.7)	110	(41.5)	0.579 (0.299–1.120)	0.102	0.839 (0.362–1.942)	0.682
CT + CC	175	(91.6)	233	(87.9)	0.666 (0.354–1.252)	0.204	0.934 (0.404–2.157)	0.872
CT	95	(49.7)	110	(41.5)	1.0 (reference)	-	-	-
TT + CC	96	(50.3)	155	(58.5)	1.394 (0.959–2.028)	0.081	1.138 (0.707–1.832)	0.593
C	255	(66.8)	356	(67.2)	1.0 (reference)	-	-	-
T	127	(33.2)	174	(32.8)	0.981 (0.742–1.298)	0.895	-	-
**TaqI** **rs731236**								
TT	65	(34.0)	102	(38.5)	1.0 (reference)	-	-	-
TC	102	(53.4)	113	(42.6)	0.706 (0.468–1.064)	0.096	0.645 (0.381–1.094)	0.104
CC	24	(12.6)	50	(18.9)	1.328 (0.745–2.366)	0.336	1.356 (0.647–2.839)	0.420
TC + CC	126	(66.0)	163	(61.5)	0.824 (0.559–1.216)	0.329	0.778 (0.475–1.274)	0.319
CC	24	(12.6)	50	(18.9)	1.0 (reference)	-	-	-
TC	102	(53.4)	113	(42.6)	0.532 (0.305–0.927)	**0.025**	0.481 (0.241–0.959)	**0.038**
TC + TT	167	(87.4)	215	(81.1)	0.618 (0.365–1.047)	0.072	0.570 (0.293–1.107)	0.097
TC	102	(53.4)	113	(42.6)	1.0 (reference)	-	-	-
CC + TT	89	(46.6)	152	(57.4)	1.542 (1.060–2.241)	**0.023**	1.697 (1.053–2.733)	**0.030**
T	232	(60.7)	317	(59.8)	1.0 (reference)	-	-	-
C	150	(39.3)	213	(40.2)	1.039 (0.794–1.360)	0.779	-	-
**BsmI rs1544410**								
GG	59	(30.9)	90	(34.0)	1.0 (reference)	-	-	-
GA	109	(57.1)	119	(44.9)	0.716 (0.471–1.088)	0.117	0.713 (0.418–1.218)	0.216
AA	23	(12.0)	56	(21.1)	1.596 (0.888–2.868)	0.117	1.481 (0.705–3.115)	0.300
GA + AA	132	(69.1)	175	(66.0)	0.869 (0.583–1.295)	0.490	0.851 (0.513–1.411)	0.531
AA	23	(12.0)	56	(21.1)	1.0 (reference)	-	-	-
GA	109	(57.1)	119	(44.9)	0.448 (0.259–0.778)	**0.004**	0.473 (0.239–0.936)	**0.032**
GA + GG	168	(88.0)	209	(78.9)	0.511 (0.302–0.865)	**0.011**	0.536 (0.277–1.036)	0.064
GA	109	(57.1)	119	(44.9)	1.0 (reference)	-	-	-
AA + GG	82	(42.9)	146	(55.1)	1.631 (1.121–2.373)	**0.010**	1.611 (1.002–2.592)	**0.049**
G	227	(59.4)	299	(56.4)	1.0 (reference)	-	-	-
A	155	(40.6)	231	(43.6)	1.131 (0.866–1.477)	0.364	-	-

OR, Odds Ratio; CI, Confidence Interval * Adjusted for age and sex. Bold values indicate statistical significance.

**Table 3:** Genotypic and allelic frequency distribution analysis of the *VDR* SNPs between Migraine without Aura (MwoA) cases and controls.

	MwoA (N = 109)		Controls (N = 265)		OR (95%CI)	*p*	OR_adj_ (95%CI) *	*p_adj_* *
	*n*	*(%)*	*n*	*(%)*				
**FokI** **rs2228570**								
CC	41	(37.6)	123	(46.4)	1.0 (reference)	-	-	-
CT	59	(54.1)	110	(41.5)	0.621 (0.387–0.999)	**0.049**	0.894 (0.491–1.627)	0.714
TT	9	(8.3)	32	(12.1)	1.185 (0.522–2.690)	0.684	1.291 (0.436–3.822)	0.645
CT + TT	68	(62.4)	142	(53.6)	0.696 (0.441–1.099)	0.119	0.929 (0.523–1.648)	0.800
TT	9	(8.3)	32	(12.1)	1.0 (reference)	-	-	-
CT	59	(54.1)	110	(41.5)	0.524 (0.235–1.172)	0.112	0.683 (0.238–1.963)	0.479
CT + CC	100	(91.7)	233	(87.9)	0.655 (0.302–1.423)	0.283	0.718 (0.256–2.018)	0.530
CT	59	(54.1)	110	(41.5)	1.0 (reference)	-	-	-
TT + CC	50	(45.9)	155	(58.5)	1.663 (1.061–2.605)	**0.026**	1.193 (0.676–2.105)	0.543
C	141	(64.7)	356	(67.2)	1.0 (reference)	-	-	-
T	77	(35.3)	174	(32.8)	0.895 (0.642–1.247)	0.512	-	-
**TaqI** **rs731236**								
TT	39	(35.8)	102	(38.5)	1.0 (reference)	-	-	-
TC	59	(54.1)	113	(42.6)	0.732 (0.451–1.189)	0.207	0.731 (0.395–1.354)	0.319
CC	11	(10.1)	50	(18.9)	1.738 (0.821–3.679)	0.146	1.935 (0.759–4.932)	0.167
TC + CC	70	(64.2)	163	(61.5)	0.890 (0.560–1.415)	0.623	0.916 (0.513–1.635)	0.767
CC	11	(10.1)	50	(18.9)	1.0 (reference)	-	-	-
TC	59	(54.1)	113	(42.6)	0.421 (0.204–0.870)	**0.017**	0.378 (0.157–0.908)	**0.030**
TC + TT	98	(89.9)	215	(81.1)	0.483 (0.241–0.967)	**0.037**	0.427 (0.183–0.997)	**0.049**
TC	59	(54.1)	113	(42.6)	1.0 (reference)	-	-	-
CC + TT	50	(45.9)	152	(57.4)	1.587 (1.014–2.486)	**0.043**	1.652 (0.938–2.907)	0.082
T	137	(62.8)	317	(59.8)	1.0 (reference)	-	-	-
C	81	(37.2)	213	(40.2)	1.136 (0.821–1.573)	0.440	-	-
**BsmI rs1544410**								
GG	37	(33.9)	90	(34.0)	1.0 (reference)	-	-	-
GA	62	(56.9)	119	(44.9)	0.789 (0.483–1.289)	0.344	0.853 (0.457–1.591)	0.617
AA	10	(9.2)	56	(21.1)	2.302 (1.062–4.993)	**0.032**	2.154 (0.836–5.555)	0.112
GA + AA	72	(66.1)	175	(66.0)	0.999 (0.624–1.600)	0.997	1.049 (0.581–1.893)	0.874
AA	10	(9.2)	56	(21.1)	1.0 (reference)	-	-	-
GA	62	(56.9)	119	(44.9)	0.343 (0.164–0.718)	**0.003**	0.374 (0.156–0.898)	**0.028**
GA + GG	99	(90.8)	209	(78.9)	0.377 (0.185–0.770)	**0.006**	0.399 (0.170–0.934)	**0.034**
GA	62	(56.9)	119	(44.9)	1.0 (reference)	-	-	-
AA + GG	47	(43.1)	146	(55.1)	1.618 (1.032–2.538)	**0.035**	1.511 (0.860–2.654)	0.151
G	136	(62.4)	299	(56.4)	1.0 (reference)	-	-	-
A	82	(37.6)	231	(43.6)	1.281 (0.927–1.771)	0.133	-	-

OR, Odds Ratio; CI, Confidence Interval * Adjusted for age and sex. Bold values indicate statistical significance.

**Table 4:** Frequency distribution analysis of the 3 *VDR* SNPs haplotypes in migraine patients and controls.

Haplotypes				Case Frequency n (%)		Control Frequency n (%)		OR (95% CI)	*p*-Value
	*FokI*	*TaqI*	*BsmI*						
**H1**	C	T	G	151.29	(0.396)	216.90	(0.409)	0.929 (0.708–1.218)	0.592
**H2**	C	C	A	94.70	(0.248)	131.74	(0.249)	0.982 (0.723–1.334)	0.909
**H3**	T	T	G	73.68	(0.193)	82.10	(0.155)	1.289 (0.911–1.825)	0.151
**H4**	T	C	A	53.26	(0.139)	81.26	(0.153)	0.883 (0.607–1.285)	0.516

**Table 5:** Frequency distribution analysis of the 3 *VDR* SNPs haplotypes in migraine without aura (MwoA) patients and controls.

Haplotypes				MwoA Frequency n (%)		Control Frequency n (%)		OR (95% CI)	*p*-Value
	*FokI*	*TaqI*	*BsmI*						
**H1**	C	T	G	90.05	(0.413)	216.90	(0.409)	0.997 (0.721–1.378)	0.985
**H2**	C	C	A	45.90	(0.211)	131.74	(0.249)	0.793 (0.541–1.162)	0.234
**H3**	T	T	G	43.92	(0.201)	82.10	(0.155)	1.361 (0.906–2.045)	0.137
**H4**	T	C	A	33.07	(0.152)	81.26	(0.153)	0.975 (0.628–1.513)	0.909

**Table 6:** Analysis of the association of the three VDR variants with clinical features in migraineurs.

***FokI* rs2228570**	**CC**	**CT**	**TT**	***p***
Migraineurs subjects (N = 191)	80	95	16	-
Age at onset (years)	20.73 ± 8.85 19 (7–52)	19.34 ± 8.40 17 (5–47)	20.19 ± 5.37 20 (5–29)	0.179
Attack frequency (days/month)	11.09 ± 8.60 8 (0.25–30)	10.81 ± 9.02 8 (1–30)	12.18 ± 8.89 10 (0.25–25)	0.773
***TaqI* rs731236**	**TT**	**TC**	**CC**	***p***
Migraineurs subjects (N = 191)	65	102	24	-
Age at onset (years)	21.12 ± 9.27 19 (9–52)	19.34 ± 7.68 18 (5–47)	19.67 ± 8.73 18 (6–40)	0.698
Attack frequency (days/month)	11.32 ± 9.31 7 (1–30)	10.47 ± 8.33 8 (0.25–30)	12.71 ± 9.41 11 (1–30)	0.598
***BsmI* rs1544410**	**GG**	**GA**	**AA**	***p***
Migraineurs subjects (N = 191)	59	109	23	-
Age at onset (years)	22.25 ± 9.94 19 (12–52)	18.78 ± 7.10 17 (5–45)	19.91 ± 8.84 18 (6–40)	0.200
Attack frequency (days/month)	10.81 ± 8.95 7 (1–30)	10.92 ± 8.68 8 (0.25–30)	12.17 ± 9.24 10 (1–30)	0.766

Data are presented as mean ± SD and median (min–max).

## Data Availability

Available upon reasonable request.
